# A Computationally Efficient MUSIC Algorithm with an Enhanced DOA Estimation Performance for a Crossed-Dipole Array

**DOI:** 10.3390/s25113469

**Published:** 2025-05-30

**Authors:** Hao Nan, Xiaofeng Ma, Yubing Han, Weixing Sheng

**Affiliations:** School of Electronic and Optical Engineering, Nanjing University of Science and Technology, Nanjing 210094, China; nanhao@njust.edu.cn (H.N.); hanyb@njust.edu.cn (Y.H.); shengwx@njust.edu.cn (W.S.)

**Keywords:** real-valued operation, sum–difference covariance matrix, computationally efficient

## Abstract

In this article, an improved real-valued dimension-reduction MUSIC (IRDR-MUSIC) algorithm is proposed for a crossed-dipole array. Initially, conjugate symmetry of the spatial component in the manifold vector is derived such that two real-valued matrices for the sum and difference covariance are constructed, which consist of the real and imaginary parts of the complex covariance matrix respectively. However, sum and difference covariance matrices with information loss would deteriorate the performance. Thus, given that the sum and difference covariance matrices have an identical null space, a joint sum–difference covariance matrix combining both the sum and difference covariance matrices is constructed, which includes the complete information of a complex covariance matrix. Accordingly, a computationally efficient IRDR-MUSIC algorithm with an enhanced performance is proposed. Compared with the existing dimension-reduction MUSIC algorithm, the proposed IRDR-MUSIC algorithm greatly reduce the complexity reduction almost without any performance loss since singular-value decomposition of the joint sum–difference covariance matrix operates in the real-valued domain, and only half of the range of the spatial spectrum search is required. Furthermore, the proposed IRDR-MUSIC algorithm outperforms the state-of-art complex-valued, symmetry-compressed, dimension-reduction MUSIC algorithm in both its multi-target resolution and computational efficiency. Numerical simulations and analyses verify the superiority of the proposed IRDR-MUSIC algorithm.

## 1. Introduction

Estimating the direction-of-arrival (DOA) of electromagnetic sources has been extensively applied in diverse areas, including communication systems [1,2,3], satellite navigation [4,5,6], and sonar [7] and radar systems [8,9,10,11]. In these application areas, the efficient methods integrated into real-time localization systems for estimating the electromagnetic propagation properties of the received signals are important. Antonello [12] proposed a new technique for estimating the angle-of-arrival (AoA) of multiple signals via a phase interferometric approach, which was low-complexity and could be implemented directly in the digital hardware. Then, he designed a fully digital synchronous architecture for AoA estimation based on phase interferometry, which was lightweight and capable of AoA estimation in real time [13]. On the other hand, subspace-based methods such as multiple signal classification (MUSIC) [14], estimation of the signal parameters via rotational invariance techniques (ESPRIT) [15], and subspace fitting [16] are also major research orientations for DOA estimation. Among these methods, the MUSIC algorithm [17,18] has garnered significant attention due to its superior parameter estimation performance and the independence of the array structure. However, its practical application in real-time systems is constrained by the extensive computational complexity inherent in the steps of spectrum search, the construction of the covariance matrix, and singular-value decomposition (SVD).

The spectrum search in MUSIC accounts for its major computational complexity, especially in case where MUSIC is applied to electromagnetic vector sensor arrays (EMVSAs), which require high-dimensional spatial–polarization spectrum searches. Therefore, it is the priority to reduce the complexity of the spectrum search [19,20,21,22]. Dimension-reduction processing is an effective solution. Wang [19] used twice one-dimensional (1-D) spatial spectrum searches rather than using once 2-D search to estimate both the elevation and azimuth angles of the signals from an L-shaped array. Then, Lan [20] simplified 4-D spatial–polarization spectrum searches into 2-D spatial and polarization spectrum searches by separating the array manifold vector into spatial and polarization components. Moreover, reducing the spectrum search range reduction is an alternative method. Yan [21] proposed a compressed MUSIC algorithm for scalar sensor arrays, employing the intersection of the noise-like subspace cluster, in which only a sector of the field of view (FOV) needed to be searched for all of the signals in the whole FOV to be determined. Nan [22] promoted the spectrum compression technique for EMVSAs, while only symmetry compression is available, i.e., the range of the spatial spectrum search is halved.

Apart from the spectrum search, reducing the complexity reduction of constructing covariance matrix and the SVD involving substantial complex-valued multiplication calculations are also crucial. On the one hand, many research works have focused on the noise subspace estimation methods without SVD, such as the propagator method [23,24] and fast subspace decomposition [25,26]. Akkar [23] proposed a propagator method in which the linear computation was used to derive the noise subspace rather than SVD such that the computatioal complexity was reduced. On the other hand, given that one complex multiplication is equivalent to four real multiplications, the transformation of complex-valued operations into real-valued domain would mitigate their complexity [27,28,29,30]. Huarng [27] proposed a unitary-MUSIC algorithm using a unitary matrix to convert the complex covariance matrix into a real-valued matrix. However, unitary-MUSIC is only suitable for centro-symmetric arrays since the complex covariance matrix must be hermitian persymmetric. To remedy this problem, Yan [30] proposed a real-valued MUSIC (RV-MUSIC) algorithm without restrictions on the array structure. However, the performance of RV-MUSIC algorithm for DOA estimation is deteriorated because only the real or the imaginary part of the complex covariance matrix is utilized.

In this article, an improved, real-valued dimension-reduction MUSIC (IRDR-MUSIC) algorithm is proposed. First, real-valued sum and difference covariance matrices are constructed, employing the conjugate symmetry property of the spatial component in the manifold vector. Then, a joint sum-difference covariance matrix is constructed by combining both sum and difference covariance matrices that share an identical null space, and thus the IRDR-MUSIC algorithm is proposed. Compared with the dimension-reduction MUSIC (DR-MUSIC) algorithm in [20], IRDR-MUSIC possesses a comparable performance and has a significant advantage in terms of its computational complexity because only half of the range of the spatial spectrum search is required and the SVD operates in the real-valued domain. Furthermore, IRDR-MUSIC is superior to the complex-valued symmetry-compressed dimension-reduction MUSIC (SC-DR-MUSIC) algorithm obtained by [22] in terms of both its multi-target resolution and computational efficiency.

The rest of this article is organized as follows. In Section 2, The linear crossed-dipole array (LCDA) model is constructed. In Section 3, the IRDR-MUSIC algorithm is proposed and the computational complexity is analyzed. Numerical simulations and analysis are performed in Section 4 with conclusions in Section 5.

Notations: ${\left(\cdot \right)}^{T}$, ${\left(\cdot \right)}^{*}$, and ${\left(\cdot \right)}^{H}$ denote the transpose, conjugate, and conjugate transpose of a matrix or a vector, respectively; C refers to the complex domain, $E\left\{\cdot \right\}$ is the statistical expectation operator, and ⊗ represents the Kronecker product; $I_{N}$ is the identity matrix of size *N* and $\mathrm{diag}\left(\cdot \right)$ denotes diagonal matrix; $span\left(\cdot \right)$ and ∩ represent the spanned space and its intersection, respectively; $Re\left(\cdot \right)$ and $Im\left(\cdot \right)$ represent the real and imaginary part of matrix, respectively; |·| denotes the determinant of matrix.

## 2. Model Construction and CRB

**Figure 1:**
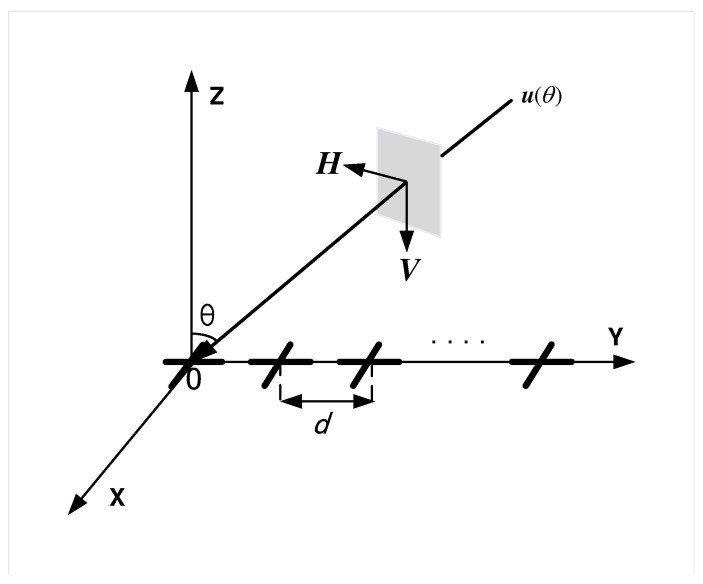
Schematic representation of a planar EM wave from θ impinging on an LCDA located at *y* axis.

As shown in Figure 1, a planar electromagnetic wave received by an LCDA located at *y* axis consists of an electric field E and a magnetic field H, orthogonal to the unit propagation direction vector $u\left(\theta \right)$, where $\theta \in \left(-\pi /2,\pi /2\right)$ is elevation angle. The polarization characteristic of E can be expressed by the polarization vector p(γ,η) [31] as

(1) $$p\left(\gamma ,\eta \right)=\left[\begin{array}{c} \xi_{H}\\ \xi_{V} \end{array}\right]=\left[\begin{array}{c} \cos\gamma \\ \sin\gamma e^{j\eta } \end{array}\right],$$

where $\xi_{H}$ and $\xi_{V}$ represent the horizontally and vertically polarization components, respectively. $\gamma \in \left[0,\pi /2\right]$ is the auxiliary polarization angle and $\eta \in \left(-\pi ,\pi \right]$ is the polarization phase difference.

Assume *K* far-field narrowband signals impinge on an LCDA composed of *M* crossed-dipole with inter-element spacing d=λ/2, where λ is signal wavelength, $y={\left[y_{1},\ldots ,y_{M}\right]}^{T}={\left[\lambda /2,\ldots ,M\lambda /2\right]}^{T}$ is coordinate vector, $\left(\theta_{1},\gamma_{1},\eta_{1}\right),\ldots ,\left(\theta_{K},\gamma_{K},\eta_{K}\right)$ are the DOA and polarization parameters of these *K* signals. Then, the LCDA output vector $x\left(t\right)\in C^{2M\times 1}$ is

(2) x(t)=As(t)+n(t),

where $s\left(t\right)=[s_{1}\left(t\right),\ldots ,s_{K}\left(t\right){]}^{T}\in C^{K\times 1}$ is signal vector and n(t) represents an additive white gaussian noise vector with zero mean and covariance $\sigma^{2}I_{2M}$, $\sigma^{2}$ is noise power. $A=[a\left(\theta_{1},\gamma_{1},\eta_{1}\right),\ldots ,a\left(\theta_{K},\gamma_{K},\eta_{K}\right)]\in C^{2M\times K}$ is the spatial–polarization manifold matrix of LCDA.

Then, according to $\left(B_{1}B_{2}\right)⊗\left(B_{3}B_{4}\right)=\left(B_{1}⊗B_{3}\right)\left(B_{2}⊗B_{4}\right)$, where $B_{i},i=1,\ldots ,4$ denotes arbitrary matrix, $a\left(\theta ,\gamma ,\eta \right)$ can be rewritten as

(3) $$\begin{array}{cc} a(\theta ,\gamma ,\eta ) & =a_{s}\left(\theta \right)⊗G\left(\theta \right)p\left(\gamma ,\eta \right)\\ & =a_{G}\left(\theta \right)p\left(\gamma ,\eta \right) \end{array},$$

where $a_{G}\left(\theta \right)=a_{s}\left(\theta \right)⊗G\left(\theta \right)\in C^{2M\times 2}$, $a_{s}\left(\theta \right)=[e^{\frac{2\pi }{\lambda }y_{1}\sin\theta },\ldots ,e^{j\frac{2\pi }{\lambda }y_{M}\sin\theta }{]}^{T}\in C^{M\times 1}$ is the spatial steering vector of LCDA, G(θ) is 1-D response matrix of crossed-dipole as [32]

(4) $$G\left(\theta \right)=\left(\begin{array}{cc} -1 & 0\\ 0 & \cos\theta \end{array}\right).$$

Accordingly, the array output covariance matrix $R\in C^{2M\times 2M}$ is

(5) $$\begin{array}{cc} R & =E\{x\left(t\right)x^{H}\left(t\right)\}\\ \; & =AR_{s}A^{H}+\sigma^{2}I_{2M}\\ \; & =U_{s}\Lambda_{s}U_{s}^{H}+U_{n}\Lambda_{n}U_{n}^{H} \end{array},$$

where $R_{s}$ is signal covariance matrix; $\Lambda_{s}=diag\left(\lambda_{1},\ldots ,\lambda_{K}\right)$, $\Lambda_{n}=diag\left(\lambda_{K+1},\ldots ,\lambda_{2M}\right)$ with the eigenvalues of R ranked in descending order as $\lambda_{1}\geq \ldots \geq \lambda_{K}>\lambda_{K+1}=\ldots =\lambda_{2M}$; $U_{s}$ and $U_{n}$ are signal and noise subspaces consisting of the eigenvectors associated with $\Lambda_{s}$ and $\Lambda_{n}$, respectively.

In practice, the sampled covariance matrix $\hat{R}$ is estimated from *L* snapshots of the array output vector as

(6) $$\hat{R}=\frac{1}{L}\sum_{l=1}^{L}x\left[l\right]x^{H}\left[l\right].$$

If the number of snapshots *L* is sufficiently large, the deterministic Cramer-Rao Bound (CRB) serving as the lower bound of error covariance for any unbiased estimation is

(7) CRB(α)=σ22L[Re(Δ⊙P^s)]−1,

where α=(θ,γ,η), $\Delta ={\dot{a}}^{H}\Pi_{a}^{⊥}\dot{a}$, ${\hat{P}}_{s}=I_{3}⊗{\hat{R}}_{s}$, ${\hat{R}}_{s}=\frac{1}{L}\sum_{t_{l}=1}^{L}s\left(t_{l}\right)s^{H}\left(t_{l}\right)$. $\dot{a}$ is the derivative vector of a(α) in (3), $\Pi_{a}^{⊥}=I_{2M}-a{\left(a^{H}a\right)}^{-1}a^{H}$.

## 3. Proposed Algorithm

This section starts by reviewing the DR-MUSIC algorithm in [20] and the SC-DR-MUSIC algorithm obtained by [22]. On this basis, an IRDR-MUSIC algorithm with lower complexity is proposed by constructing a real-valued sum-difference covariance matrix, which containing both real and imaginary parts of original complex covariance matrix.

### 3.1. DR-MUSIC Algorithm

According to the orthogonality between the manifold vector corresponding to the *k*th signal from $\left(\theta_{k},\gamma_{k},\eta_{k}\right)$ and the ideal noise subspace, we have

(8) $$U_{n}^{H}a(\theta_{k},\gamma_{k},\eta_{k})=0.$$

Substituting (3) into (8) as

(9) $$U_{n}^{H}a_{G}\left(\theta_{k}\right)p(\gamma_{k},\eta_{k})=0.$$

Thus, the spatial–polarization spectrum function of MUSIC is obtained as

(10) $$F\left(\theta ,\gamma ,\eta \right)=\frac{1}{a^{H}\left(\theta ,\gamma ,\eta \right){\hat{U}}_{n}{\hat{U}}_{n}^{H}a\left(\theta ,\gamma ,\eta \right)},$$

where ${\hat{U}}_{n}$ is the estimated noise subspace of $\hat{R}$ in (6).

Equation (9) indicates that the column vectors of $U_{n}^{H}a_{G}\left(\theta_{k}\right)$ are linearly dependent, i.e., the column rank of $U_{n}^{H}a_{G}\left(\theta_{k}\right)$ is less than 2. Consequently, $a_{G}^{H}\left(\theta_{k}\right)U_{n}U_{n}^{H}a_{G}\left(\theta_{k}\right)$ is a rank-deficient matrix implying that

(11) $$|a_{G}^{H}\left(\theta_{k}\right)U_{n}U_{n}^{H}a_{G}\left(\theta_{k}\right)|=0.$$

Accordingly, the 1-D DOA estimator of DR-MUSIC algorithm [20] is obtained as

(12) $$f_{DR}\left(\theta \right)=\frac{1}{|a_{G}^{H}\left(\theta \right){\hat{U}}_{n}{\hat{U}}_{n}^{H}a_{G}\left(\theta \right)|}.$$

Finally, by substituting the estimated DOA of *k*th signal ${\hat{\theta }}_{k}$ obtained from (12) into (10), its polarization parameters $\left({\hat{\gamma }}_{k},{\hat{\eta }}_{k}\right)$ can be estimated by a 2-D polarization spectrum search. Although the DR-MUSIC algorithm exhibits lower complexity compared with MUSIC, its overall complexity remains substantial.

Then, by employing the symmetry-compressed technique applying for EMVSAs [21,22], the 1-D spatial spectrum function of SC-DR-MUSIC algorithm is obtained as

(13) $$f_{SC}\left(\theta \right)=\frac{1}{|a_{G}^{H}\left(\theta \right){\hat{U}}_{n}{\hat{U}}_{n}^{H}{{\hat{U}}_{n}}^{*}{\hat{U}}_{n}^{T}a_{G}\left(\theta \right)|}.$$

$a_{G}\left(\theta \right)$ in (13) can be expressed in blocks as

(14) $$a_{G}\left(\theta \right)=\left[\begin{array}{c} a_{g1}\left(\theta \right)\\ \ldots \\ a_{gM}\left(\theta \right) \end{array}\right],$$

where $a_{gm}\left(\theta \right)=\left[\begin{array}{cc} -e^{jm\pi \sin\theta } & 0\\ 0 & \cos\theta e^{jm\pi \sin\theta } \end{array}\right]\in C^{2\times 2}$.

Considering sine function is odd function, it has $a_{s}^{*}\left(\theta \right)=a_{s}\left(-\theta \right)$. Accordingly, it derives that

(15) $$a_{gm}^{*}\left(\theta \right)=a_{gm}\left(-\theta \right).$$

That is,

(16) $$a_{G}^{*}\left(\theta \right)=a_{G}\left(-\theta \right).$$

Due to the fact that the determinants of a matrix and its transpose are identical, the null spectrum of SC-DR-MUSIC algorithm in (13) can be rewritten as

(17) $$\begin{array}{c} |a_{G}^{H}\left(\theta \right){\hat{U}}_{n}{\hat{U}}_{n}^{H}{\hat{U}}_{n}^{*}{\hat{U}}_{n}^{T}a_{G}\left(\theta \right)|\\ =|a_{G}^{T}\left(\theta \right){\left({\hat{U}}_{n}{\hat{U}}_{n}^{H}{\hat{U}}_{n}^{*}{\hat{U}}_{n}^{T}\right)}^{T}{\left(a_{G}^{H}\left(\theta \right)\right)}^{T}|\\ =|a_{G}^{T}\left(\theta \right){\hat{U}}_{n}{\hat{U}}_{n}^{H}{\hat{U}}_{n}^{*}{\hat{U}}_{n}^{T}a_{G}^{*}\left(\theta \right)| \end{array}.$$

Then, substituting (16) into (17), it derives that

(18) $$\begin{array}{c} |a_{G}^{H}\left(\theta \right){\hat{U}}_{n}{\hat{U}}_{n}^{H}{\hat{U}}_{n}^{*}{\hat{U}}_{n}^{T}a_{G}\left(\theta \right)|=|a_{G}^{H}\left(-\theta \right){\hat{U}}_{n}{\hat{U}}_{n}^{H}{\hat{U}}_{n}^{*}{\hat{U}}_{n}^{T}a_{G}\left(-\theta \right)| \end{array},$$

which indicates that $f_{SC}\left(\theta \right)=f_{SC}\left(-\theta \right)$, i.e., the spatial spectrum function of SC-DR-MUSIC algorithm is symmetric with respect to θ=0. Therefore, the range of spatial spectrum search of SC-DR-MUSIC algorithm is halved resulting that it is more efficient than DR-MUSIC algorithm.

### 3.2. IRDR-MUSIC Algorithm

#### 3.2.1. 1-D DOA Estimation

In this subsection, the IRDR-MUSIC algorithm is proposed for 1-D DOA estimation.

According to (16), the conjugate of (8) can be expressed as

(19) $${\left(U_{n}^{*}\right)}^{H}a^{*}\left(\theta_{k},\gamma_{k},\eta_{k}\right)={\left(U_{n}^{*}\right)}^{H}a_{G}^{*}\left(\theta_{k}\right)p^{*}\left(\gamma_{k},\eta_{k}\right)={\left(U_{n}^{*}\right)}^{H}a_{G}\left(-\theta_{k}\right)p^{*}\left(\gamma_{k},\eta_{k}\right)=0,$$

which implies that $U_{n}^{*}$ is the orthogonal subspace of $a^{*}\left(\theta_{k},\gamma_{k},\eta_{k}\right)$.

Define the intersection of the span spaces of $U_{n}$ and $U_{n}^{*}$ as $\mathrm{span}\left(G_{n}\right)=span\left(U_{n}\right)\cap span\left(U_{n}^{*}\right)$, according to (9) and (19), there surely exist

$$\left\{ \begin{array}{cc} G_{n}^{H}a_{G}\left(\theta_{k}\right)p\left(\gamma_{k},\eta_{k}\right)=G_{n}^{H}A\left(\theta_{k},\gamma_{k},\eta_{k}\right)=0, & (20a)\\ G_{n}^{H}a_{G}\left(-\theta_{k}\right)p*\left(\gamma_{k},\eta_{k}\right)=G_{n}^{H}A^{*}\left(\theta_{k},\gamma_{k},\eta_{k}\right)=0. & (20b) \end{array}\right.$$

Then, R and $R^{*}$ are right-multiplied by $G_{n}$ as

(21) $$RG_{n}=AR_{s}A^{H}G_{n}+\sigma^{2}G_{n}=\sigma^{2}G_{n},$$

(22) $$R^{*}G_{n}=A^{*}{R_{s}}^{*}{\left(A^{*}\right)}^{H}G_{n}+\sigma^{2}G_{n}=\sigma^{2}G_{n},$$

where $\sigma^{2}=\frac{1}{2M-K}\sum_{i=K+1}^{2M}\lambda_{i}$.

By adding and substracting (20) and (21) with each other, we derive

(23) $$\left(R+R^{*}\right)G_{n}=2Re\left(R\right)G_{n}=2\sigma^{2}G_{n},$$

(24) $$\left(R-R^{*}\right)G_{n}=2jIm\left(R\right)G_{n}=0.$$

Then, (23) and (24) are rewritten as

(25) $$R_{sum}G_{n}=\left(Re\left(R\right)-\sigma^{2}I_{2M}\right)G_{n}=0,$$

(26) $$R_{dif}G_{n}=Im\left(R\right)G_{n}=0,$$

where

(27) $$R_{sum}=\frac{\left(R+R^{*}-2\sigma^{2}I_{2M}\right)}{2}=Re\left(R\right)-\sigma^{2}I_{2M},$$

(28) $$R_{dif}=\frac{\left(R-R^{*}\right)}{2j}=Im\left(R\right).$$

However, it is proved that if the noise subspace of ${\hat{R}}_{sum}$ or ${\hat{R}}_{dif}$ containing only real or imaginary part of $\hat{R}$ is utilized, the parameter estimation performance would deteriorate.

The sampled covariance matrix $\hat{R}$ in (6) can be expressed as

(29) $$\hat{R}=\frac{1}{L}\hat{X}{\hat{X}}^{H},$$

where $\hat{X}\in C^{2M\times L}$ is sampled array output matrix. Then, $\hat{R}$ is rewritten as

(30) $$\begin{array}{cc} \hat{R} & =\frac{1}{L}\hat{X}{\hat{X}}^{H}\\ & =\frac{1}{L}\left\{\left[Re\left(\hat{X}\right)+jIm\left(\hat{X}\right)\right]\left[{Re}^{T}\left(\hat{X}\right)-j{Im}^{T}\left(\hat{X}\right)\right]\right\}\\ & =\frac{1}{L}\left\{Re\left(\hat{X}\right){Re}^{T}\left(\hat{X}\right)+Im\left(\hat{X}\right){Im}^{T}\left(\hat{X}\right)\right\}+\frac{j}{L}\left\{{Re}^{T}\left(\hat{X}\right)Im\left(\hat{X}\right)-Re\left(\hat{X}\right){Im}^{T}\left(\hat{X}\right)\right\} \end{array}.$$

It can be seen that the real part of $\hat{R}$ only contains the self-correlation components of the real and imaginary parts of $\hat{X}$, the imaginary part of $\hat{R}$ only contains the cross-correlation components of the real and imaginary parts of $\hat{X}$. Therefore, the utilization of either the real or imaginary part of $\hat{R}$ would inevitably deteriorate the performance. In other words, both the real and imaginary parts of $\hat{R}$ should be utilized together to avoid the performance loss.

It is noticed from (25) and (26) that the null spaces of $R_{sum}$ and $R_{dif}$ are identical to $span(G_{n})$. Accordingly, the 1-D sum–difference covariance matrix $R_{sd}$ is constructed by combining $R_{sum}$ and $R_{dif}$ together as $R_{sd}=\left[\begin{array}{c} R_{sum}\\ R_{dif} \end{array}\right]\in C^{4M\times 2M}$ such that

(31) $$R_{sd}G_{n}=\left[\begin{array}{c} R_{sum}\\ R_{dif} \end{array}\right]G_{n}=\left[\begin{array}{c} Re\left(R\right)-\sigma^{2}I_{2M}\\ Im(R) \end{array}\right]G_{n}=0.$$

In practice, the SVD is performed on the 1-D sampled sum–difference covariance matrix ${\hat{R}}_{sd}$ to obtain the real-valued noise subspace ${\hat{G}}_{n}\in C^{2M\times (2M-2K)}$. Substituting ${\hat{G}}_{n}$ for ${\hat{U}}_{n}$ in (12), the 1-D DOA estimator of IRDR-MUSIC algorithm is obtained as

(32) $$f_{IRDR}\left(\theta \right)=\frac{1}{|a_{G}^{H}\left(\theta \right){\hat{G}}_{n}{\hat{G}}_{n}^{H}a_{G}\left(\theta \right)|}.$$

It can be seen from (20) that both $|a_{G}^{H}\left(\theta_{k}\right)G_{n}G_{n}^{H}a_{G}\left(\theta_{k}\right)|$ and $|a_{G}^{H}\left(-\theta_{k}\right)G_{n}G_{n}^{H}a_{G}\left(-\theta_{k}\right)|$ are equal to zero. Therefore, for the *k*th signal, there exist two peaks at the positions of ${\hat{\theta }}_{k}$ and $-{\hat{\theta }}_{k}$ in the 1-D spatial spectrum of IRDR-MUSIC algorithm within the whole FOV, i.e., $\theta \in (-90^{\circ },90^{\circ })$. Substituting ${\hat{\theta }}_{k}$ and $-{\hat{\theta }}_{k}$ into (11), the true DOA of the *k*th signal can be determined as long as the left side of (11) approaches to zero. It indicates that only half of the range of FOV, i.e., $\theta \in (0,90^{\circ })$ is required to search for the proposed IRDR-MUSIC algorithm to determine all the signals within the whole FOV. Meanwhile, the performance of IRDR-MUSIC algorithm is enhanced since ${\hat{R}}_{sd}$ contains both the real and imaginary parts of $\hat{R}$. Furthermore, it is noted that M>K is required to guarantee that $span({\hat{G}}_{n})$ is not empty.

The detailed steps of the proposed IRDR-MUSIC algorithm for 1-D DOA estimation are summarized in Algorithm 1.
**Algorithm 1** The proposed IRDR-MUSIC algorithm for 1-D DOA estimation**Input:** The LCDA received signal vector x(t) with *L* snapshots. **Steps:**
   Step 1:Construct the sampled complex covariance matrix $\hat{R}$ and its conjugate ${\hat{R}}^{*}$, respectively.   Step 2:Obtain the sampled sum and difference covariance matrices ${\hat{R}}_{sum}$ and ${\hat{R}}_{dif}$ from (27) and (28).   Step 3:Construct the sampled 1-D sum–difference covariance matrix as ${\hat{R}}_{sd}=\left[\begin{array}{c} {\hat{R}}_{sum}\\ {\hat{R}}_{dif} \end{array}\right]$, and perform the SVD to obtain its noise subspace ${\hat{G}}_{n}$.   Step 4:Substitute ${\hat{G}}_{n}$ into the 1-D DOA estimator of the IRDR-MUSIC algorithm in (32), and perform the spatial spectrum search within $\theta \in (0,90^{\circ })$ to obtain the estimated DOA ${\hat{\theta }}_{k}$.   Step 5:Substitute ${\hat{\theta }}_{k}$ and $-{\hat{\theta }}_{k}$ into $|a_{G}^{H}\left(\theta \right){\hat{U}}_{n}{\hat{U}}_{n}^{H}a_{G}\left(\theta \right)|$ to determine the true DOA of the *k*th signal as follows.
(1)If $|a_{G}^{H}\left({\hat{\theta }}_{k}\right){\hat{U}}_{n}{\hat{U}}_{n}^{H}a_{G}\left({\hat{\theta }}_{k}\right)|$ approaches to zero and fails for $a_{G}\left(-{\hat{\theta }}_{k}\right)$, the true DOA of the *k*th signal is ${\hat{\theta }}_{k}$.(2)If $|a_{G}^{H}\left(-{\hat{\theta }}_{k}\right){\hat{U}}_{n}{\hat{U}}_{n}^{H}a_{G}\left(-{\hat{\theta }}_{k}\right)|$ approaches to zero and fails for $a_{G}\left({\hat{\theta }}_{k}\right)$, the true DOA of the *k*th signal is $-{\hat{\theta }}_{k}$.(3)If both $\left(a_{G}^{H}\left({\hat{\theta }}_{k}\right){\hat{U}}_{n}{\hat{U}}_{n}a_{G}\left({\hat{\theta }}_{k}\right)\right)$ and $\left(a_{G}^{H}\left(-{\hat{\theta }}_{k}\right){\hat{U}}_{n}{\hat{U}}_{n}a_{G}\left(-{\hat{\theta }}_{k}\right)\right)$ approcah to zero, the true signal DOAs are ${\hat{\theta }}_{k}$ and $-{\hat{\theta }}_{k}$.

**Output:** The estimated 1-D true DOAs of signals.

#### 3.2.2. 2-D DOA Estimation

In this subsection, the IRDR-MUSIC algorithm is extended to the planar crossed-dipole array (PCDA) for 2-D DOA estimation.

When *K* far-field narrowband signals impinge on the PCDA from $\left(\theta_{1},\varphi_{1},\gamma_{1},\eta_{1}\right),\ldots ,\left(\theta_{K},\varphi_{K},\gamma_{K},\eta_{K}\right)$, respectively, the PCDA output vector $x_{p}\left(t\right)\in C^{2M\times 1}$ is

(33) $$x_{p}\left(t\right)=A_{p}s\left(t\right)+n\left(t\right),$$

where $A_{p}=[a\left(\theta_{1},\varphi_{1},\gamma_{1},\eta_{1}\right),\ldots ,a\left(\theta_{K},\varphi_{K},\gamma_{K},\eta_{K}\right)]\in C^{2M\times K}$ is the spatial–polarization manifold matrix of the PCDA. The manifold vector a(θ,φ,γ,η) is

(34) $$\begin{array}{cc} a(\theta ,\varphi ,\gamma ,\eta ) & =a_{s}\left(\theta ,\varphi \right)⊗G\left(\theta ,\varphi \right)p\left(\gamma ,\eta \right)\\ & =a_{G}\left(\theta ,\varphi \right)p\left(\gamma ,\eta \right) \end{array},$$

where $a_{G}\left(\theta ,\varphi \right)=a_{s}\left(\theta ,\varphi \right)⊗G\left(\theta ,\varphi \right)\in C^{2M\times 2}$, $a_{s}\left(\theta ,\varphi \right)=[e^{\frac{2\pi }{\lambda }d(x_{1}u+y_{1}v)},\ldots ,e^{j\frac{2\pi }{\lambda }d(x_{M}u+y_{M}v)}{]}^{T}\in C^{M\times 1}$ is the spatial steering vector of PCDA, u=sinθcosφ and v=sinθsinφ are direction cosines, $\left(x_{m},y_{m}\right)$ is the coordinate of *m*th element in the PCDA, G(θ,φ) is the 2-D response matrix of crossed-dipole as [22]

(35) $$G\left(\theta ,\varphi \right)=\left(\begin{array}{cc} -\sin\varphi & \cos\theta \cos\varphi \\ \cos\varphi & \cos\theta \sin\varphi \end{array}\right).$$

Then, the PCDA output covariance matrix $R_{p}\in C^{2M\times 2M}$ is

(36) $$\begin{array}{cc} R_{p} & =A_{p}R_{s}A_{p}^{H}+\sigma^{2}I_{2M}\\ & =V_{s}F_{s}V_{s}^{H}+V_{n}F_{n}V_{n}^{H} \end{array},$$

where $V_{s}$ and $V_{n}$ are signal and noise subspaces of $R_{p}$, respectively.

Similar to (14), the blocked $a_{G}\left(\theta ,\varphi \right)$ in (34) can be written as

(37) $$a_{G}\left(\theta ,\varphi \right)=\left[\begin{array}{c} a_{g1}\left(\theta ,\varphi \right)\\ \ldots \\ a_{gM}\left(\theta ,\varphi \right) \end{array}\right],$$

where $a_{gm}\left(\theta ,\varphi \right)=\left[\begin{array}{cc} -\sin\varphi e^{j\pi (x_{m}u+y_{m}v)} & \cos\theta \cos\varphi e^{j\pi (x_{m}u+y_{m}v)}\\ \cos\varphi e^{j\pi (x_{m}u+y_{m}v)} & \cos\theta \sin\varphi e^{j\pi (x_{m}u+y_{m}v)} \end{array}\right]$.

Given that sinθcos(φ+π)=−sinθcosφ and sinθsin(φ+π)=−sinθsinφ, thus, $a_{s}\left(\theta ,\varphi +\pi \right)=e^{-j\pi (x_{m}u+y_{m}v)}=a_{s}^{*}\left(\theta ,\varphi \right)$ is established. Accordingly, it derives that

(38) $$a_{gm}^{*}\left(\theta ,\varphi \right)=a_{gm}\left(\theta ,\varphi +\pi \right),$$

that is,

(39) $$a_{G}^{*}\left(\theta ,\varphi \right)=a_{G}\left(\theta ,\varphi +\pi \right).$$

According to the orthogonality between the manifold vector corresponding to the *k*th signal from $\left(\theta_{k},\varphi_{k},\gamma_{k},\eta_{k}\right)$ and the ideal noise subspace $V_{n}$, it has

(40) $$V_{n}^{H}a(\theta_{k},\varphi_{k},\gamma_{k},\eta_{k})=0.$$

The conjuate of (40) is expressed as

(41) $${\left(V_{n}^{*}\right)}^{H}a^{*}\left(\theta_{k},\varphi_{k},\gamma_{k},\eta_{k}\right)={\left(V_{n}^{*}\right)}^{H}a_{G}^{*}\left(\theta_{k},\varphi_{k}\right)p^{*}\left(\gamma_{k},\eta_{k}\right)={\left(V_{n}^{*}\right)}^{H}a_{G}\left(\theta_{k},\varphi_{k}+\pi \right)p^{*}\left(\gamma_{k},\eta_{k}\right)=0,$$

which indicates that $V_{n}^{*}$ is the orthogonal subspace of $a^{*}\left(\theta_{k},\varphi_{k},\gamma_{k},\eta_{k}\right)$, and the column vectors of ${\left(V_{n}^{*}\right)}^{H}a_{G}\left(\theta_{k},\varphi_{k}+\pi \right)$ are also linearly dependent.

Define the intersection of the span spaces of $V_{n}$ and $V_{n}^{*}$ as $\mathrm{span}\left(Q_{n}\right)=span\left(V_{n}\right)\cap span\left(V_{n}^{*}\right)$, thus, according to (40) and (41), there surely exist

$$\{\begin{array}{cc} Q_{n}^{H}a_{G}\left(\theta_{k},\varphi_{k}\right)p\left(\gamma_{k},\eta_{k}\right)=Q_{n}^{H}A_{p}\left(\theta_{k},\varphi_{k},\gamma_{k},\eta_{k}\right)=0, & (42a)\\ Q_{n}^{H}a_{G}\left(\theta_{k},\varphi_{k}+\pi \right)p*\left(\gamma_{k},\eta_{k}\right)=Q_{n}^{H}A_{p}^{*}\left(\theta_{k},\varphi_{k},\gamma_{k},\eta_{k}\right)=0. & (42b) \end{array}$$

Then, $R_{p}$ and $R_{p}^{*}$ are right-multiplied by $Q_{n}$ as

(43) $$R_{p}Q_{n}=A_{p}R_{s}A_{p}^{H}Q_{n}+\sigma^{2}Q_{n}=\sigma^{2}Q_{n},$$

(44) $$R_{p}^{*}Q_{n}=A_{p}^{*}{R_{s}}^{*}{\left(A_{p}^{*}\right)}^{H}Q_{n}+\sigma^{2}Q_{n}=\sigma^{2}Q_{n}.$$

By adding and substracting (43) and (44) with each other, we obtain

(45) $$\left(R_{p}+R_{p}^{*}\right)Q_{n}=2Re\left(R_{p}\right)Q_{n}=2\sigma^{2}Q_{n},$$

(46) $$\left(R_{p}-R_{p}^{*}\right)Q_{n}=2jIm\left(R_{p}\right)Q_{n}=0.$$

Then, (45) and (46) are rewritten as

(47) $$R_{psum}Q_{n}=\left(Re\left(R_{p}\right)-\sigma^{2}I_{2M}\right)Q_{n}=0,$$

(48) $$R_{pdif}Q_{n}=Im\left(R_{p}\right)Q_{n}=0,$$

where

(49) $$R_{psum}=\frac{\left(R_{p}+R_{p}^{*}-2\sigma^{2}I_{2M}\right)}{2}=Re\left(R_{p}\right)-\sigma^{2}I_{2M},$$

(50) $$R_{pdif}=\frac{\left(R_{p}-R_{p}^{*}\right)}{2j}=Im\left(R_{p}\right).$$

It is noticed from (47) and (48) that the null spaces of $R_{psum}$ and $R_{pdif}$ are identical to $span(Q_{n})$. Similar to the construction of $R_{sd}$ in (31), the 2-D sum–difference covariance matrix $R_{psd}$ is constructed by combining $R_{psum}$ and $R_{pdif}$ together as $R_{psd}=\left[\begin{array}{c} R_{psum}\\ R_{pdif} \end{array}\right]\in C^{4M\times 2M}$ such that

(51) $$R_{psd}Q_{n}=\left[\begin{array}{c} R_{psum}\\ R_{pdif} \end{array}\right]Q_{n}=\left[\begin{array}{c} Re\left(R_{p}\right)-\sigma^{2}I_{2M}\\ Im(R_{p}) \end{array}\right]Q_{n}=0.$$

In practice, the SVD is performed on the sampled 2-D sum–difference covariance matrix ${\hat{R}}_{psd}$ to obtain its real-valued noise subspace ${\hat{Q}}_{n}\in C^{2M\times (2M-2K)}$. Substituting ${\hat{Q}}_{n}$ for ${\hat{U}}_{n}$ in (12), the 2-D DOA estimator of the IRDR-MUSIC algorithm is obtained as

(52) $$f_{IRDR}\left(\theta .\varphi \right)=\frac{1}{|a_{G}^{H}\left(\theta ,\varphi \right){\hat{Q}}_{n}{\hat{Q}}_{n}^{H}a_{G}\left(\theta ,\varphi \right)|}.$$

Equations (42a) and (42b) demonstrate that both $|a_{G}^{H}\left(\theta_{k},\varphi_{k}\right)Q_{n}Q_{n}^{H}a_{G}\left(\theta_{k},\varphi_{k}\right)|$ and $|a_{G}^{H}\left(\theta_{k},\varphi_{k}+\pi \right)Q_{n}Q_{n}^{H}a_{G}\left(\theta_{k},\varphi_{k}+\pi \right)|$ are equal to zero. Therefore, for the *k*th signal, there exist two peaks at the positions of $\left({\hat{\theta }}_{k},{\hat{\varphi }}_{k}\right)$ and $\left({\hat{\theta }}_{k},{\hat{\varphi }}_{k}+\pi \right)$ in the 2-D spatial spectrum of the proposed IRDR-MUSIC algorithm within $\theta \in (0^{\circ },90^{\circ })$, $\varphi \in (0^{\circ },360^{\circ })$. Then, substituting $\left({\hat{\theta }}_{k},{\hat{\varphi }}_{k}\right)$ and $\left({\hat{\theta }}_{k},{\hat{\varphi }}_{k}+\pi \right)$ into the 2-D DOA estimator of DR-MUSIC algorithm, the true DOA of the *k*th signal can be determined. Therefore, only half range of the FOV, i.e., $\theta \in (0^{\circ },90^{\circ })$, $\varphi \in (0^{\circ },180^{\circ })$ is required to search for the IRDR-MUSIC algorithm to determine all signals within the whole FOV.

The detailed steps of the proposed IRDR-MUSIC algorithm for 2-D DOA estimation are summarized in Algorithm 2.
**Algorithm 2** The proposed IRDR-MUSIC algorithm for 2-D DOA estimation**Input:** The PCDA received signal vector $x_{p}\left(t\right)$ with *L* snapshots. **Steps:**
   Step 1:Construct the sampled complex covariance matrix ${\hat{R}}_{p}$ and its conjugate ${\hat{R}}_{p}^{*}$, respectively.   Step 2:Obtain the sampled sum and difference covariance matrices ${\hat{R}}_{psum}$ and ${\hat{R}}_{pdif}$ from (49) and (50).   Step 3:Construct the sampled 2-D sum–difference covariance matrix as ${\hat{R}}_{psd}=\left[\begin{array}{c} {\hat{R}}_{psum}\\ {\hat{R}}_{pdif} \end{array}\right]$, and perform the SVD to obtain its noise subspace ${\hat{Q}}_{n}$.   Step 4:Substitute ${\hat{Q}}_{n}$ into the 2-D DOA estimator of IRDR-MUSIC algorithm in (52), and perform the spatial spectrum search within $\theta \in (0,90^{\circ })$, $\varphi \in (0^{\circ },180^{\circ })$ to obtain the estimated DOA $\left({\hat{\theta }}_{k},{\hat{\varphi }}_{k}\right)$.   Step 5:Substitute $\left({\hat{\theta }}_{k},{\hat{\varphi }}_{k}\right)$ and $\left({\hat{\theta }}_{k},{\hat{\varphi }}_{k}+180^{\circ }\right)$ into $|a_{G}^{H}\left(\theta ,\varphi \right){\hat{V}}_{n}{\hat{V}}_{n}^{H}a_{G}\left(\theta ,\varphi \right)|$ to determine the true DOA of the *k*th signal as follows.
(1)If $|a_{G}^{H}\left({\hat{\theta }}_{k},{\hat{\varphi }}_{k}\right){\hat{V}}_{n}{\hat{V}}_{n}^{H}a_{G}\left({\hat{\theta }}_{k},{\hat{\varphi }}_{k}\right)|$ approaches to zero and fails for $a_{G}\left({\hat{\theta }}_{k},{\hat{\varphi }}_{k}+180^{\circ }\right)$, the true DOA of the *k*th signal is $\left({\hat{\theta }}_{k},{\hat{\varphi }}_{k}\right)$.(2)If $|a_{G}^{H}\left({\hat{\theta }}_{k},{\hat{\varphi }}_{k}+180^{\circ }\right){\hat{V}}_{n}{\hat{V}}_{n}^{H}a_{G}\left({\hat{\theta }}_{k},{\hat{\varphi }}_{k}+180^{\circ }\right)|$ approaches to zero and fails for $a_{G}\left({\hat{\theta }}_{k},{\hat{\varphi }}_{k}\right)$, the true DOA of the *k*th signal is $\left({\hat{\theta }}_{k},{\hat{\varphi }}_{k}+180^{\circ }\right)$.(3)If both $a_{G}^{H}\left({\hat{\theta }}_{k},{\hat{\varphi }}_{k}+180^{\circ }\right){\hat{V}}_{n}{\hat{V}}_{n}a_{G}\left({\hat{\theta }}_{k},{\hat{\varphi }}_{k}+180^{\circ }\right)$ and $a_{G}^{H}\left({\hat{\theta }}_{k},{\hat{\varphi }}_{k}\right){\hat{V}}_{n}{\hat{V}}_{n}a_{G}\left({\hat{\theta }}_{k},{\hat{\varphi }}_{k}\right)$ approcah to zero, the true signal DOAs are $\left({\hat{\theta }}_{k},{\hat{\varphi }}_{k}\right)$ and $\left({\hat{\theta }}_{k},{\hat{\varphi }}_{k}+180^{\circ }\right)$.

**Output:** The estimated 2-D true DOAs of signals.


### 3.3. Computational Complexity Analysis

The computational complexities of the DR-MUSIC [20], SC-DR-MUSIC [22], Unitary-MUSIC [27], and IRDR-MUSIC algorithms are mainly composed of three parts: construction of covariance matrix, SVD, and spatial spectrum search, which are summarized in Table 1, where *J* is the number of spatial spectrum search points and J≫M. Given that the step of spectrum search accounts for the major complexity, the computational complexities of the SC-DR-MUSIC and IRDR-MUSIC are reduced compared with that of the DR-MUSIC and Unitary-MUSIC because the range of spatial spectrum search is halved. Furthermore, the IRDR-MUSIC exhibits lower complexity than the SC-DR-MUSIC and the Unitary-MUSIC is more efficient than DR-MUSIC because both IRDR-MUSIC and Unitary-MUSIC operate in the real-valued domain.

## 4. Simulations Results and Discussion

In this section, simulations are performed to validate the superiority of the proposed IRDR-MUSIC algorithm. Given that the performance comparison results of 1-D and 2-D DOA estimations are consistent, only 1-D DOA estimation of the proposed IRDR-MUSIC algorithm for the LCDA is exhibited throughout the simulations. In following simulations, the LCDA consists of M=10 crossed-dipoles with d=λ/2, elemental SNR is 0dB, the number of snapshots L=512 and the sample grid interval is $0.1^{\circ }$, except for additional declaration.

### 4.1. Spatial Spectrum Estimation

**Figure 2:**
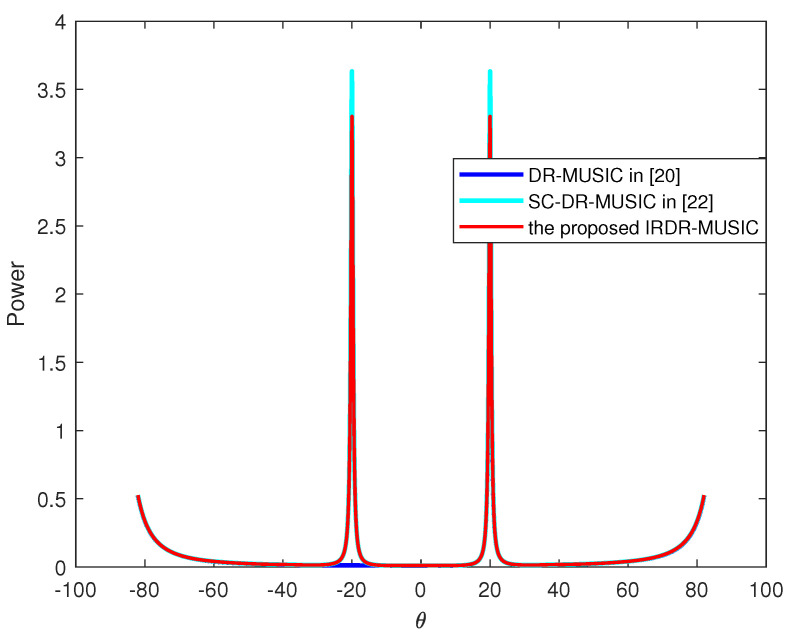
Spatial spectrum of the DR-MUSIC, SC-DR-MUSIC, and the proposed IRDR-MUSIC algorithms when the signal impinges from $\left(\theta ,\gamma ,\eta \right)=\left(20^{\circ },25^{\circ },60^{\circ }\right)$.

Assume a signal from $\left(\theta ,\gamma ,\eta \right)=\left(20^{\circ },25^{\circ },60^{\circ }\right)$ impinges on the LCDA. The spatial spectrums of the DR-MUSIC, SC-DR-MUSIC, and the proposed IRDR-MUSIC algorithms are shown in Figure 2. It can be seen that only single spectrum peak exists in the spatial spectrum of the DR-MUSIC algorithm associated with the true DOA of the signal. While an additional spectrum peak exhibits at the symmetric position of true DOA in both the spatial spectrums of SC-DR-MUSIC and IRDR-MUSIC algorithms. It demonstrates that only half of FOV is required to search for the SC-DR-MUSIC and IRDR-MUSIC algorithms. Then, the true DOA of the signal can be determined by substituting the estimated $\hat{\theta }$ and $-\hat{\theta }$ into the left side of (11) and justifying whether $|a_{G}^{H}\left(\hat{\theta }\right){\hat{G}}_{n}{\hat{G}}_{n}^{H}a_{G}\left(\hat{\theta }\right)|$ or $|a_{G}^{H}\left(-\hat{\theta }\right){\hat{G}}_{n}{\hat{G}}_{n}^{H}a_{G}\left(-\hat{\theta }\right)|$ approaches to zero.

### 4.2. Estimation Accuracy

Root mean square error (RMSE) is defined as

(53) $$RMSE=\sqrt{\frac{1}{WK}\sum_{w=1}^{W}\sum_{k=1}^{K}{\left({\hat{\theta }}_{k,w}-\theta_{k}\right)}^{2}},$$

where ${\hat{\theta }}_{k,w}$ is the estimated DOA of the *k*th signal in the *w*th run, *K* is the number of signals. In following simulations, W=1000 Monte Carlo trials is used.

**Figure 3:**
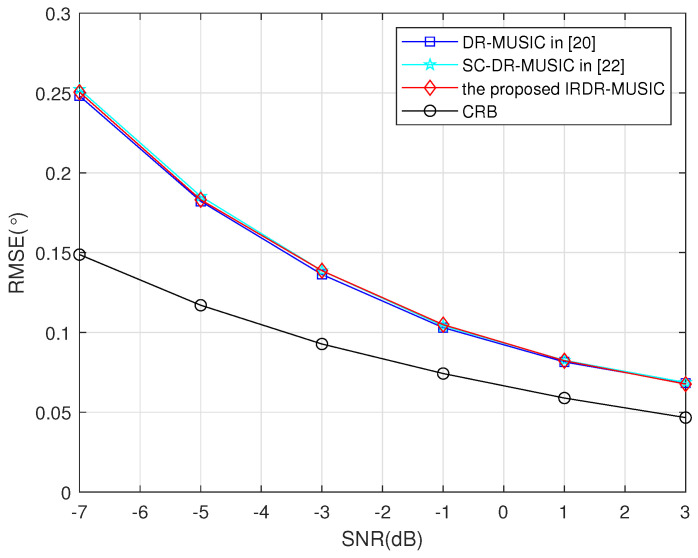
RMSEs of the DR-MUSIC, SC-DR-MUSIC, and IRDR-MUSIC algorithms vs. SNR when a signal impinges from $\left(\theta ,\gamma ,\eta \right)=\left(30^{\circ },70^{\circ },45^{\circ }\right)$.

**Figure 4:**
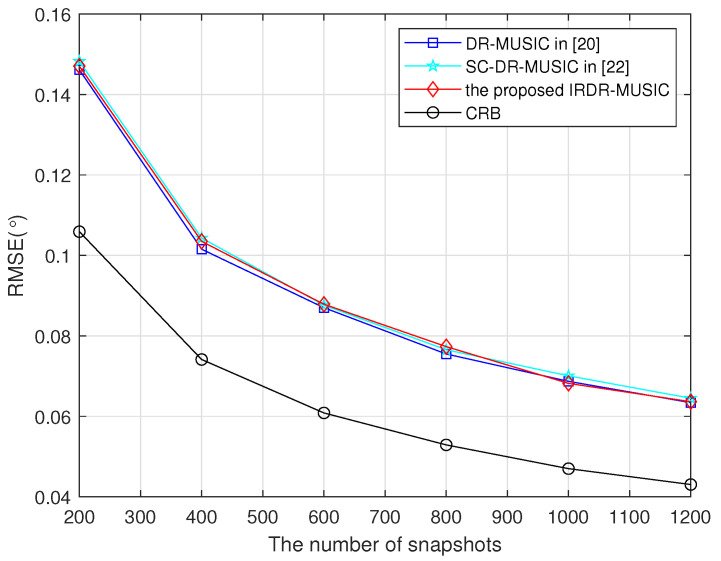
RMSEs of the DR-MUSIC, SC-DR-MUSIC, and IRDR-MUSIC algorithms vs. the number of snapshots when a signal impinges from $\left(\theta ,\gamma ,\eta \right)=\left(30^{\circ },70^{\circ },45^{\circ }\right)$.

Assume a signal from $\left(\theta ,\gamma ,\eta \right)=\left(30^{\circ },70^{\circ },45^{\circ }\right)$ impinges on the LCDA. RMSEs of the DR-MUSIC, SC-DR-MUSIC, and IRDR-MUSIC algorithms vs. SNR and the number of snapshots are shown in Figure 3 and Figure 4, respectively. It shows that the DOA estimation accuracies of the DR-MUSIC, SC-DR-MUSIC, and IRDR-MUSIC algorithms are comparable because the covariance matrices used in such three algorithms contain the complete real and imaginary parts of R.

### 4.3. Multi-Target Resolution

Probability of resolution is used to evaluate the multi-target resolution. If there exist

(54) $$\frac{f\left(\theta_{1}\right)+f\left(\theta_{2}\right)}{2}>f\left(\frac{\theta_{1}+\theta_{2}}{2}\right),$$

two sources from $\theta_{1}$ and $\theta_{2}$ are regarded as spatially resolved.

**Figure 5:**
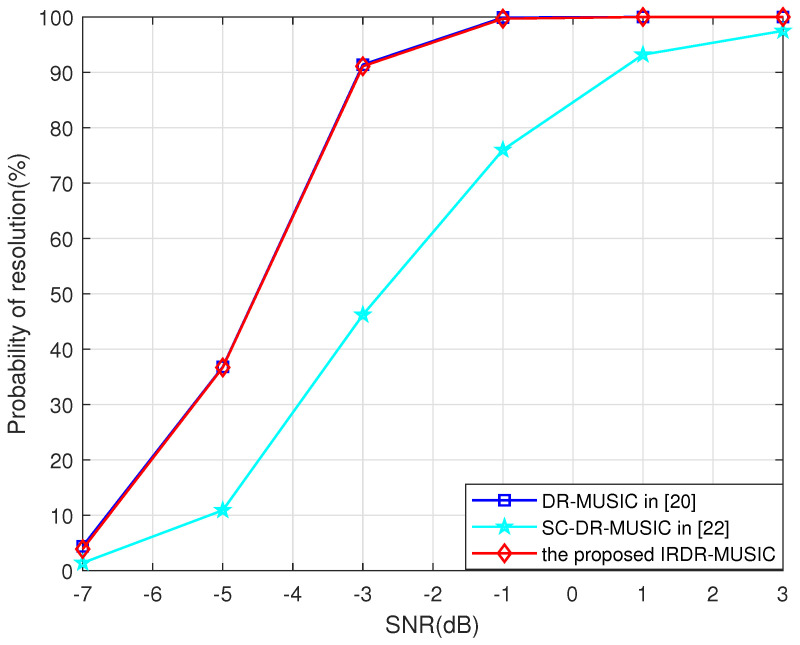
Probabilities of resolution for the DR-MUSIC, SC-DR-MUSIC, and IRDR-MUSIC algorithms vs. SNR when two signals impinge from $\left(\theta_{1},\gamma_{1},\eta_{1}\right)=\left(20^{\circ },45^{\circ },30^{\circ }\right)$ and $\left(\theta_{2},\gamma_{2},\eta_{2}\right)=\left(26^{\circ },50^{\circ },40^{\circ }\right)$.

**Figure 6:**
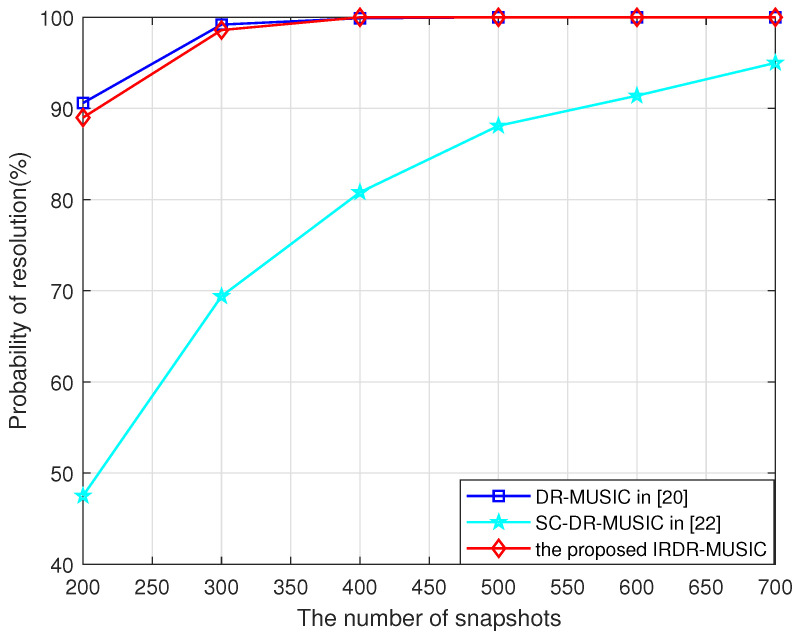
Probabilities of resolution for the DR-MUSIC, SC-DR-MUSIC, and IRDR-MUSIC algorithms vs. the number of snapshots when two signals impinge from $\left(\theta_{1},\gamma_{1},\eta_{1}\right)=\left(20^{\circ },45^{\circ },30^{\circ }\right)$ and $\left(\theta_{2},\gamma_{2},\eta_{2}\right)=\left(26^{\circ },50^{\circ },40^{\circ }\right)$.

Figure 5 and Figure 6 give the probabilities of resolution for the DR-MUSIC, SC-DR-MUSIC, and IRDR-MUSIC algorithms vs. SNR and the number of snapshots when two signals impinge on the LCDA from $\left(\theta_{1},\gamma_{1},\eta_{1}\right)=\left(20^{\circ },45^{\circ },30^{\circ }\right)$ and $\left(\theta_{2},\gamma_{2},\eta_{2}\right)=\left(26^{\circ },50^{\circ },40^{\circ }\right)$. It is obvious that the multi-target resolution of the proposed IRDR-MUSIC algorithm is comparable to that of the DR-MUSIC algorithm, and superior to that of the SC-DR-MUSIC algorithm.

### 4.4. Running Time

**Figure 7:**
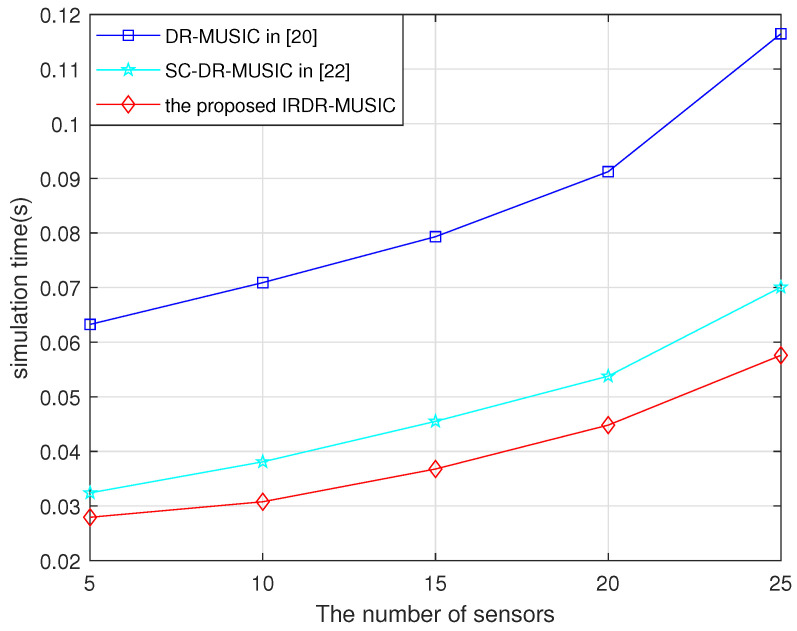
Running times of the DR-MUSIC, SC-DR-MUSIC, and IRDR-MUSIC algorithms vs. the number of sensors.

Here, the running times of the DR-MUSIC, SC-DR-MUSIC, and IRDR-MUSIC algorithms vs. the number of sensors are recorded in Figure 7. The CPU of hardware platform is Inter I5-11400H with 16 GB RAM. It is shown that the time consumptions of SC-DR-MUSIC and IRDR-MUSIC algorithms are both greatly reduced compared with that of the DR-MUSIC algorithm because the range of spatial spectrum search is halved. Moreover, the time consumption of the IRDR-MUSIC algorithm is less than that of the SC-DR-MUSIC algorithm since the complex-valued operations are converted to the real-valued domain. The simulation results in Figure 7 are consistent with the computational complexity analysis in Table 1.

In summary, compared with the DR-MUSIC algorithm [20], the computational complexity of the proposed IRDR-MUSIC algorithm is greatly reduced since the SVD of joint sum–difference covariance matrix is operated in the real-valued domain and the range of the spatial spectrum search is halved. Furthermore, their performances are comparable, because the joint sum–difference covariance matrix contains the complete real and imaginary parts of complex covariance matrix. Furthermore, the IRDR-MUSIC algorithm operating in real-valued domain has advantages in terms of both multi-target resolution and efficiency compared with the SC-DR-MUSIC algorithm obtained by [22].

## 5. Conclusions

In this article, a computationally efficient IRDR-MUSIC algorithm with enhanced performance is proposed. First, the sum and difference covariance matrices are constructed employing only the real and imaginary parts of the complex covariance matrix, respectively. However, the utilization of either the sum or difference covariance matrix suffering from the information loss would deteriorate the parameter estimation performance. Then, benefitting from the sum and difference covariance matrices sharing the identical null subspace, a sum–difference covariance matrix combining both the real and imaginary parts of the complex covariance matrix is constructed for the proposed IRDR-MUSIC algorithm with enhanced performance. The estimation accuracy and multi-target resolution of the IRDR-MUSIC algorithm are comparable to that of the DR-MUSIC algorithm, while its computational complexity is greatly reduced. Furthermore, the IRDR-MUSIC algorithm has advantages in terms of both the multi-target resolution and computational efficiency compared with the SC-DR-MUSIC algorithm. It is noted that the proposed IRDR-MUSIC algorithm is not only suitable for the crossed-dipole array. It can also apply to other electromagnetic vector sensor arrays, such as the COLD array consisting of two orthogonal dipole and loop, as long as the conjugate symmetry property of the spatial component in array manifold vector is satisfied.

## Figures and Tables

**Table 1:** Computational complexity comparison.

Algorithms	Construction of Covariance Matrix	SVD	Spatial Spectrum Search
DR-MUSIC in [20]	$L{\left(2M\right)}^{2}$	$O(8M^{3})$	$O\left(J^{3}\right)$
SC-DR-MUSIC in [22]	$L{\left(2M\right)}^{2}$	$O(8M^{3})$	$O\left({\left(J/2\right)}^{3}\right)$
Unitary-MUSIC in [27]	$L{\left(2M\right)}^{2}$	$O(8M^{3})/4$	$O\left({\left(J\right)}^{3}\right)$
the proposed IRDR-MUSIC	$L{\left(2M\right)}^{2}$	$O(16M^{3})/4$	$O\left({\left(J/2\right)}^{3}\right)$

## Data Availability

The original contributions presented in this study are included in the article. Further inquiries can be directed to the corresponding author.

## Abbreviations

The following abbreviations are used in this manuscript:

DOA	direction-of-arrival
SVD	singular value decomposition
EMVSA	electromagnetic vector sensor array
FOV	field of view
LCDA	linear crossed-dipole array
PCDA	planar crossed-dipole array
MUSIC	multiple signal classification
DR-MUSIC	dimension-reduction MUSIC
SC-DR-MUSIC	symmetry-compressed dimension-reduction MUSIC
IRDR-MUSIC	improved real-valued dimension-reduction MUSIC
